# Effects of corn supplementation on meat quality and fatty acid composition of Dorper lambs fed PKC-Urea treated rice straw

**DOI:** 10.1186/s12917-019-1976-8

**Published:** 2019-07-08

**Authors:** O. A. Saeed, A. Q. Sazili, H. Akit, M. Ebrahimi, A. R. Alimon, A. A. Samsudin

**Affiliations:** 1 Department of Animal Science, Faculty of Agriculture, Universiti Putra Malaysia, 43400 Serdang, Selangor Malaysia; 2 Institute of Tropical Agriculture Universiti Putra Malaysia, 43400 Serdang, Selangor Malaysia; 3 Department of Veterinary Preclinical Sciences Faculty of Veterinary, Universiti Putra Malaysia, 43400 Serdang, Selangor Malaysia; 4 Faculty of Animal Science, Universitas Gadjah Mada, Yogjakarta, Indonesia; 5 Department of Animal Production Faculty of Agriculture, University Of Anbar, Anbar, Iraq

**Keywords:** Palm Kernel Cake, Meat Quality, Fatty Acid, Corn, Dorper Lambs

## Abstract

**Background:**

The increasing costs of feed has subsequently increased the costs of production of livestock, thereby decreasing the profit margin of this sector. The utilization of agro-industrial by-products has to some extent substitute some of the corn grains and soyabean meal, commonly used in animal feeds. In Malaysia, palm kernel cake (PKC) is a by-product of the oil palm industry and is frequently used to supply both crude protein (14–16% CP) and energy (11 MJ/kg) in ruminants. The energy and protein content are adequate for maintenance in the majority of ruminants. However, highly available energy supplementation is known to improve growth performance and protein deposition. This study was carried out to determine the effect on the quality of meat and fatty acid composition of the *semitendinosus* (ST), *supraspinatus* (SS), and *longissimus lumborum* (LL) muscles of Dorper lambs by including corn as an energy source in a basal diet of PKC urea-treated rice straw.

**Results:**

The results show that the LL muscle-drip loss was greater in animals supplemented with 5% corn compared to the other groups. Higher pH values of SS and LL muscles were observed in animals supplemented with 5 and 10% corn. Furthermore, the L* value of ST muscle was increased in lambs fed on 5% corn while, reduced in those fed on 0% corn, but the a* and b* values were not significantly different in the treatment groups. The fatty acid composition of the SS muscles showed that lambs fed on 10% corn had higher levels of sum PUFA n-3 compared to those fed on 0% corn. The concentration of C18:1trans11 and CLA c12 t10 in ST muscle from the lambs fed on supplemented diets were higher than those of the controls.

**Conclusion:**

This study has concluded the supplementation of corn as a source of energy into a PKC urea-treated rice straw-based diet increased the PUFA concentrations of muscles as compared to control groups.

## Background

The increasing use of unconventional feedstuffs for livestock especially in Malaysia requires an evaluation of their effects on the health status of the animals and consumers. Palm kernel cake (PKC) is used as a ruminant feed in Malaysia and its constant supply throughout the year and relatively lower price, makes it a reliable source of protein for dairy and meat animals as its crude protein content adequately meets their requirements [1, 2]. Many studies have been done on the use of PKC as supplementary feed for sheep in particular and ruminants in general but none on enriching it with certain levels of corn as an energy source [3, 4]. Previous study has conducted by [5] demonstrated the diets supplemented with energy held much potential for enhancing animal performance and deposit rates. Analysis of meat quality and fatty acid composition, among others, is important in meeting demand and promoting meat science as well as to achieve an integrated quality of research on products that are more similar [6]. Therefore, since dietary factors are important in influencing meat characteristics, it is hypothesized that feeding lambs with PKC-urea treated rice straw diets supplemented with corn improves meat quality and affects fatty acid composition in the carcass. Thus, the objective of this study was to determine the effects of including corn as an energy source in the PKC-urea treated straw based diets for growing lambs, on the changes in fatty acid content and meat quality in the *semitendinosus* (ST)*, supraspinatus* (SS), and *longissimus lumborum* (LL) muscles of Dorper lambs.

## Results

### Meat quality indicators of the different muscles of the lambs

The fatty acid profiles of the experimental diets are presented in Table 1. In general, there were small variations, but not significant, in the fatty acid contents of the diets with T3 having slightly higher NFE and calculated ME contents. The results of meat drip loss, cooking loss and shear force of the various muscles from lambs fed the treatment diets are shown in Table 2. The dietary treatments did not affect (*P >* 0.05) drip loss of the ST and SS muscles, although the LL from lambs fed T3 had lower (*P <* 0.05) drip loss than the control diet. As for LL muscle drip loss, it was higher (*P <* 0.05) in lambs fed T2 (5% corn) than lambs fed the control diet. The results of cooking losses from various muscle types indicated that the ST muscle from lambs fed T3 was significantly greater (*P <* 0.05) than from those on the other treatment diets whereas the muscles of lambs on T2 had a lower cooking loss compared to those fed the control diet (*P <* 0.05). Lambs fed T2 and diets showed significant (*P <* 0.001) reductions in the percentage of SS muscles compared with the control. The cooking loss of the LL muscles showed no significant difference (*P >* 0.05) among all the treatment groups. The dietary supplementation of corn reduced the shear force value of SS muscles in both T2 and T3 (*P <* 0.001) compared to the control group. There were no significant difference in shear force values in the ST and LL muscles among the treatment groups. The inclusion of corn in the PKC-urea treated straw diets induced a significant increase (*P <* 0.05) in the pH of the ST and SS muscles of lambs fed on T2 diets and T3 diets which were lower than the control diet.. However, for the LL muscles there were no significant difference (*P >* 0.05) in the pH among the treatment groups. The results of the meat color (L*, a*, and b*) evaluation is presented in Table 3. It appeared that meat colour was affected by corn supplementation. There was an increase (*P <* 0.001) in L* in the both ST and SS muscles of lambs fed T2 while L* was reduced in the LL muscles under the same treatment. The color a* of the ST muscles was decreased (*P <* 0.01) in lambs fed T2 and T3 (Table 3) while there was no significant effects of the treatments on SS and LL muscles. There was no difference in the meat color of the SS and LL muscles among the treatment groups except that the ST muscles of lambs on T3 had higher (*P <* 0.01) b* values associated with the more yellowish color in the lean meat.

**Table 1:** Chemical and fatty acid composition of treatments

Fatty acids	Corn		
	T1 (0%)	T2 (5%)	T3 (10%)
C12:0	8.11	4.74	5.57
C14:0	5.88	6.32	3.94
C15:0	3.67	2.43	2.68
C15:1	0.63	0.26	0.73
C16:0	31.97	35.43	39.97
C16:1 n-9	0.33	0.39	0.37
C18:0	14.14	14.74	7.59
C18:1n9	31.44	31.71	36.01
C18:2n6	3.16	3.10	2.55
C18:3n-3	1.27	1.09	1.29
ΣSFA	63.78	63.68	59.77
ΣUFA	36.21	36.31	40.22
ΣMUFA	32.41	32.37	37.12
Σn-3 PUFA	1.27	1.09	1.29
Σ n-6 PUFA	3.16	3.10	2.55
Σ PUFA	4.43	4.20	3.83
n-6: n-3 Ratio	2.71	2.85	2.18
UFA:SFA	0.56	0.57	0.67
Poly:Sat Ratio	0.07	0.06	0.07

*MUFA* Monounsaturated fatty acids, *PUFA* Polyunsaturated fatty acids, *SFA* Saturated fatty acids, *UFA* Unsaturated fatty acids

**Table 2:** Effects of dietary supplementations of corn and rice straw treated with urea on meat quality in *semitendinosus, supraspinatus* and *longissimus lumborum* muscles of lambs

Muscles	Corn			*SEM*	*P*-value
	T1 (0%)	T2 (5%)	T3 (10%)		
Drip loss (%)					
ST	2.98	2.77	2.96	0.12	0.81
SS	2.60	2.08	2.21	0.15	0.43
LL	4.33^ab^	5.08^a^	3.69^b^	0.25	0.05
Cooking loss (%)					
ST	28.87^ab^	22.04^b^	31.75^a^	1.83	0.05
SS	38.50^a^	29.92^b^	31.60^b^	1.39	0.001
LL	29.91	24.15	28.93	1.40	0.21
Shear force (kg/cm^2^)					
ST	1.03	0.78	0.91	0.05	0.17
SS	0.93^a^	0.45^b^	0.46^b^	0.11	0.001
LL	1.36	0.84	1.10	0.07	0.14
pH of meat					
ST	5.82^b^	6.46^a^	5.98^b^	0.10	0.05
SS	5.76^b^	6.11^a^	5.76^ab^	0.06	0.05
LL	5.63	6.15	5.85	0.10	0.13

^a,b^Means in the same row with different superscripts are significantly different

**Table 3:** Color characteristics (L*, a*, b*) of *semitendinosus, supraspinatus* and *longissimus lumborum* muscles of lambs fed different dietary treatments

Muscles	Color	Corn			*SEM*	*P*-value
		T1 (0%)	T2 (5%)	T3 (10%)		
ST	L *	35.92^c^	41.23^a^	37.59^b^	0.81	0.001
	a *	9.55^a^	7.30^b^	9.12^ab^	0.44	0.05
	b *	9.99^a^	8.02^b^	10.26^a^	0.35	0.01
SS	L *	34.91^b^	38.49^a^	38.06^a^	0.62	0.01
	a *	10.38	10.46	10.67	0.40	0.96
	b *	12.83	12.81	12.48	0.53	0.85
LL	L *	32.68^b^	31.67^b^	39.42^a^	1.23	0.01
	a *	10.45	10.74	10.14	0.49	0.92
	b *	12.06	11.68	10.46	0.04	0.15

^a,b,c^Means in the same row with different superscripts are significantly different

### Fatty acid composition of the *supraspinatus* muscle

The results for the SS muscles indicate that C14:0 was lower for lambs fed on 5% corn and 10% corn diets (Table 4). However, there was no variation (*P >* 0.05) among treatments in the sum of SFA, and the amounts of PUFA and MUFA, also HH, AI, DFA and trans FA among the treatment groups.

**Table 4:** Effect of treatment diets on the fatty acid composition of the *supraspinatus* muscle (g/100 g total fatty acids) of lambs

Fatty acids	Corn			*SEM*	*P*-value
	T1 (0%)	T2 (5%)	T3 (10%)		
C12:0	1.44	1.72	1.86	0.14	0.55
C14:0	11.45^a^	8.72^b^	6.21^c^	0.98	0.01
C15:0	2.58	2.80	2.43	0.30	0.90
C15:1	1.31	1.16	1.94	0.33	0.69
C16:0	22.64	26.57	23.62	0.89	0.13
C16:1n-9	1.58	1.73	1.82	0.20	0.91
C18:0	27.64	27.10	26.75	0.82	0.92
C18:1n-9	17.70	16.71	17.91	0.55	0.70
C18:1trans11	1.01	1.24	1.05	0.10	0.72
C18:2 n-6	2.67	3.63	2.91	0.23	0.33
CLA c9 t11	1.23	1.41	1.63	0.11	0.40
CLA c12 t10	0.76	0.80	0.56	0.05	0.23
C18:3 n-3	0.75	0.77	0.91	0.07	0.66
C20:4 n-6	4.16	3.35	3.10	0.38	0.56
C20:5 n-3	2.15	2.51	1.50	0.32	0.56
C22:5 n-3	1.51	1.76	1.86	0.26	0.89
C22:6 n-3	2.24	1.46	1.14	0.21	0.07
Σ SFA	64.20	66.28	66.96	0.84	0.43
ΣUSFA	35.79	33.71	33.03	0.84	0.43
Σ MUFA	21.47	20.53	22.04	0.85	0.81
ΣPUFA n-3	6.66	6.51	4.36	0.67	0.34
ΣPUFA n-6	6.83	6.11	6.02	0.58	0.08
ΣPUFA	13.49	12.62	10.38	0.61	0.72
Σ trans FA	1.01	1.24	1.05	0.10	0.79
ΣCLA	1.99	2.21	2.12	0.10	0.97
n-6/n-3	1.46	1.43	1.53	0.16	0.43
USFA/SFA	0.55	0.50	0.49	0.01	0.22
PUFA/SFA	0.21	0.19	0.17	0.007	0.08
AI	1.78	1.79	2.01	0.11	0.82
DFA	63.43	60.82	59.87	1.23	0.31
HH	0.96	0.85	0.80	0.06	0.43

^a,b,c^Means in the same row with different superscripts are significantly different

The *AI* Atherogenic index, *DFA* Desirable fatty acids and the ratio of *HH* Hypocholesterolemic and hypercholesterolemic fatty acids

### Fatty acid composition of the *longissimus lumborum* muscle

Fatty acid composition of the LL muscle indicate that the lambs did not differ (*P >* 0.05) in overall fatty acid content (Table 5) when fed different treatment diets. However, the fatty acid composition of the LL muscles showed that lambs fed on T3 had higher levels of sum PUFA n-3 compared to those fed on control diet. Further, the total trans FA and PUFA/SFA appeared to be higher (*P <* 0.05) in lambs fed T3 compared with control diet. The proportions of sum PUFA n-3, sum trans FA, and PUFA/SFA were lower for lambs fed on T2 compared to those on the control diet.

**Table 5:** Effect of dietary treatments on the fatty acid composition of *longissimus lumborum* muscle (g/100 g total fatty acids) of lambs

Fatty acids	Corn			*SEM*	*P*-value
	T1 (0%)	T2 (5%)	T3 (10%)		
C12:0	2.33	1.95	1.77	0.15	0.38
C14:0	10.17	8.02	7.37	0.68	0.23
C15:0	2.20	2.14	2.05	0.31	0.98
C15:1	1.47	1.75	0.81	0.26	0.46
C16:0	25.81	26.81	25.12	1.23	0.88
C16:1n-9	1.44	1.47	1.87	0.14	0.41
C18:0	27.68	31.75	27.98	1.16	0.32
C18:1n-9	14.89	18.86	17.27	0.78	0.10
C18:1trans11	1.79	1.71	1.10	0.23	0.47
C18:2 n-6	2.25	2.00	2.14	0.23	0.92
CLA c9 t11	0.80	0.61	0.78	0.05	0.39
CLA c12 t10	1.16	0.76	0.74	0.09	0.13
C18:3 n-3	0.78	0.90	1.03	0.05	0.25
C20:4 n-6	4.14	3.32	3.82	0.37	0.73
C20:5 n-3	2.11	0.90	2.53	0.33	0.08
C22:5 n-3	2.00	1.72	2.74	0.24	0.21
C22:6 n-3	1.51	1.44	2.42	0.21	0.09
Σ SFA	67.71	70.20	64.31	1.35	0.21
ΣUSFA	32.28	29.79	35.68	1.35	0.21
Σ MUFA	19.15	20.24	20.96	1.06	0.82
ΣPUFA n-3	6.24^ab^	4.58^b^	7.94^a^	0.56	0.05
ΣPUFA n-6	6.40	5.32	5.96	0.32	0.45
ΣPUFA	12.64^ab^	9.91^b^	13.90^a^	0.71	0.05
Σ trans FA	1.33	1.71	1.10	0.23	0.63
ΣCLA	1.96	1.38	1.52	0.11	0.10
n-6/n-3	1.03	1.24	0.75	0.12	0.28
USFA/SFA	0.48	0.43	0.55	0.02	0.20
PUFA/SFA	0.18^ab^	0.14^b^	0.21^a^	0.01	0.05
AI	2.14	2.08	1.58	0.20	0.24
DFA	59.97	61.54	63.67	0.64	0.26
HH	0.77	0.75	0.96	0.05	0.25

^a,b^Means in the same row with different superscripts are significantly different. The *AI* Atherogenic index, *DFA* Desirable fatty acids and the ratio of *HH* Hypocholesterolemic and hypercholesterolemic fatty acids

### Fatty acid composition of *semitendinosus* muscle

The composition of fatty acids in the ST muscle was similar for all treatments although their levels increased slightly with the inclusion of corn in the diets (Table 6). The proportions of monounsaturated fatty acids (MUFA) and polyunsaturated fatty acids (PUFA) (C16:1, C18:1, C18:2, C18:3) differed from saturated fatty acids (SFA) (C14:0, C16:0, C18:0). The concentrations of C18:1trans11 and CLA c12 t10 content of ST muscle from the lambs fed T2 and T3 were consistently higher (*P <* 0.05) than those fed on the control diet. The highest concentration of C18:2 n-6 was found in lambs fed T2, while the lowest were observed in the control lambs. The proportions of C22:6 n-3 and ΣPUFA n-3 declined (*P <* 0.05) as the levels of corn increased in the diet. Lambs fed on T2 and T3 diets had similar (*P >* 0.05) proportions of CLA c9 t11 as those fed the control diet. Although there were no significant (*P >* 0.05) differences among the treatments in the total SFA, MUFA, n-3, and PUFA/SFA ratio in ST, the total n-6, n-6/n-3 ratio, and trans-FA were higher (*P <* 0.05) in lambs fed T3 diets compared to the other diets.

**Table 6:** Effect of treatment diets on the fatty acid composition of *semitendinosus* muscle (g/100 g total fatty acids) of lambs

Fatty acids	Corn			*SEM*	*P*-value
	T1 (0%)	T2 (5%)	T3 (10%)		
C12:0	1.54	1.51	1.15	0.10	0.29
C14:0	10.87	10.52	10.51	0.54	0.96
C15:0	2.47	2.30	2.54	0.16	0.86
C15:1	0.84	1.29	1.18	0.09	0.12
C16:0	23.90	23.28	24.20	0.42	0.72
C16:1n-9	1.72	1.35	1.59	0.12	0.51
C18:0	28.62	29.92	31.17	0.64	0.30
C18:1n-9	15.86	16.02	14.91	0.47	0.65
C18:1trans11	1.39^ab^	0.84^b^	1.98^a^	0.20	0.001
C18:2 n-6	1.97^b^	2.89^a^	1.81^b^	0.20	0.05
CLA c9 t11	1.48	1.14	0.82	0.16	0.30
CLA c12 t10	0.45^b^	0.66^a^	0.66^a^	0.04	0.05
C18:3 n-3	1.01	1.14	1.13	0.06	0.72
C20:4 n-6	3.55	3.97	4.18	0.22	0.58
C20:5 n-3	2.19	1.76	1.15	0.25	0.26
C22:5 n-3	1.19	1.51	0.93	0.19	0.55
C22:6 n-3	2.34^a^	1.53^b^	1.71^ab^	0.15	0.05
Σ SFA	67.42	67.54	69.58	0.74	0.47
ΣUSFA	32.57	32.46	30.42	0.74	0.47
Σ MUFA	19.51	19.51	19.68	0.39	0.98
ΣPUFA n-3	6.43^a^	5.56^ab^	4.57^b^	0.34	0.05
ΣPUFA n-6	5.53	6.86	5.99	0.26	0.09
ΣPUFA	11.96	12.43	10.56	0.43	0.20
Σ trans FA	1.39^ab^	0.84^b^	1.98^a^	0.20	0.001
ΣCLA	1.94	1.81	1.36	0.17	0.40
n-6/n-3	0.86^b^	1.25^a^	1.31^a^	0.08	0.01
USFA/SFA	0.48	0.48	0.44	0.01	0.48
PUFA/SFA	0.17	0.18	0.15	0.008	0.24
AI	2.12	2.08	2.23	0.22	0.89
DFA	61.20	62.38	61.59	1.78	0.89
HH	0.80	0.85	0.74	0.04	0.61

^a,b^Means in the same row with different superscripts are significantly different

The *AI* Atherogenic index, *DFA* Desirable fatty acids and the ratio of *HH* Hypocholesterolemic and hypercholesterolemic fatty acids

## Discussion

### Meat quality indicators of different muscles of lambs

There was no significant drip loss after 24 h for LL muscle of lambs fed T3 diets and this was in agreement with Yarali et al. [7] who suggested that this could be due to the fat thickness of the carcasses. A study has demonstrated that the carcasses of high and medium dietary energy groups had a low percentage of evaporation loss due to the improved fatness of their carcasses [8]. In contrast, feeding animals with oil-source diets decreased drip loss in the *semimembranosus* muscle because of a decline in protein oxidation [9]. However, this study showed that dietary corn has an effect on the cooking loss of lamb meat with the loss being the highest for the SS muscles in 10% corn. Cooking loss was affected by corn supply, which reduced the LL muscle of this parameter for both 5% corn and 10% corn (Table 2). This is favorable since meat with lower water-holding capacity will lose water resulting in a higher cooking loss. The results are in agreement with Yarali et al. [7] who showed that the various ways of cooking and muscle sections produced different values in different studies. The cooking loss (mean 26.55%) in this study was within a reasonable range. Previous research has shown the potential of using alternative feed ingredients without degrading carcass characteristics and meat quality [10, 11]. This could explain why PKC has no effect on the meat quality of lambs and that the variations among treatments may be due to the inclusion of corn in the diet. The study by Fadil [8] noted that higher cooking loss was observed in the meat of kids fed a low-energy diet while other studies suggest that a more acceptable reason was related to the pH of the meat [7]. In this study, shear force decreased significantly in 5% corn and 10% corn compared with the control group although still remaining within the acceptable limits of 3.26 kg/cm^2^ or less in terms of tenderness of the meat [12]. The increase in the body weight of animals fed on corn diets led to a reduction in the tenderness of the meat, especially in 5% corn and 10% corn. The study has stated that shear-force values decreased as weight increased [13]. Another study by Yarali et al. [7] note that the maximum shear force value was derived from the *M. semitendinosus* muscle, while the *M. longissimus dorsal* muscle offered the most tender meat. In this study, the mean pH values were from 5.63 to 6.74 at 24 h post mortem. These results are on par with the findings of [14] and within the acceptable range of lambs meat. Lambs fed on 5% corn had higher pH in the ST and SS muscles, according to [15] the pH of meat from 5.4–5.5 is characterized as normal and has the highest desirable eating properties. Previous study has done by [16] reported the meat’s tenderness was greater when pH rises from 5.5 to 6.0. A possible explanation could be the lower proteolytic activity at intermediate levels of pH (5.8 to 6.3) as it is outside the pH optima for two separate enzyme systems [17]. The pH is negatively correlated to cooking loss [18], thus changes in pH loss values observed at 24 h in the ST and SS muscles justify the decline in cooking loss of lambs fed 5% corn. In this study, the selected muscles reflected different responses to meat color with regard to dietary treatments as these authors [19, 20] repoert that diet contain on polyphenols could effect on meat color by effecting on myoglobin. The inclusion corn increased the L* of the SS and LL muscles for Dorper lambs. The obtained data agrees with Juarez et al. [21] who reported that higher L* was attributed to the slaughter weight of the Merino sheep breed. This finding shows significant negative correlation between the L* value of the meat and the pH of the muscle. However, because there were no differences in the a* color of the other muscles it is difficult to determine that diets supplemented with PKC were solely responsible for the improved stability of meat color [22]. Lambs in this study had an alert in b* of the ST muscle but had no significant values for the SS and LL muscles. These results are similar to a study conducted by [23]. The treatment differences in color appear to be due to variations in muscular myoglobin contents or in the fat content of the carcasses. As such, an increase in intramuscular fat might lead to reduced myoglobin and sarcoplasmic protein concentrations in the meat, inducing differences in the meat color [24, 25].

### Fatty acid composition of adipose tissues in lambs

The total concentrations of short, medium, and long-chain fatty acids in the SS and LL muscles were similar among treatments (Tables 4 and 5) suggesting a decline in the efficiency of the process of long-chain fatty acids absorption from the diets to muscles. These findings concur with [26] who showed that an unchanged composition of muscle fatty acids in the present study might be due to the constant stearoyl-CoA desaturase (SCD) gene expression from the high content of Cu in the PKC. However, the high concentration of PUFA in the LL muscle of animals fed on 10% corn could be associated with the PUFA content of corn which is more resistant to biohydrogenation than other cereals. The muscles of such lambs normally have high percentages of PUFA, in particular linoleic acid (18:2n-6), compared to heavier lambs [27]. The concentrations of C14:0 were reduced in the SS muscle of 5% corn and 10% corn and this is in line with the results of [28], while the addition of PKM raised the level of C12:0 and C14:0 in the diet as reflected in the concentration of fatty acids in the meat. Palm kernel cake has high concentrations of C16:0 and C16:1 (palmitoleic acid) and as a result the levels of C12:0 and C14:0 gradually increased in the SS muscles. Supplementing the diet of goats with palm oil blend decreased C14:0 and C16:0 concentrations in the SM muscles [29]. This could be attributed to the reduction in mRNA and the actions of lipogenic enzymes such as fatty acid synthesis and the acetylCoA carboxylase needed to synthesize medium-chain fatty acid [9]. However, [28] stated that the concentration of C14:0 was increased when PKM was added to the muscles of lambs. The levels of C14:0, C15:0, C15:1, and C16:0 in all the ST muscles did not vary significantly among the dietary treatments in the present experiment. These results contradict that of [4] who suggested that high SFA composition was found in the PKC. The present study did not observe any effects of PKC on the ST muscle. However, the level of C18:1trans11 and C18:2 n-6 fatty acids was elevated in the ST muscle of lambs fed the supplemented diets compared to the controls (Table 6). This is similar to the results reported by [28]. Compared to the present study it may be due to differences in the extent of ruminal biohydrogenation of C18:2n-6, with the lower biohydrogenation in our study being due to an acidic ruminal pH resulting from the high concentrate fed to the lambs. However, the rise in the concentrations of trans-vaccenic acid (and the other isomers of C18:1) in the plasma and the meat may be due to the high-lipid in the diet, which may have interfered with the rumen micro flora involved in the biohydrogenation of dietary C18:2 and C18:3 [30]. This c9, t11 C18:2 is an extremely biologically-active conjugated linoleic acid isomer, which has a positive impact on the prevention of cancer, cardiovascular disease, and diabetes, and protects the immune system [31]. The results of this study show a significant increase in CLA c12 t10 of ST muscles within control and T2 diets. This finding agrees with Ribeiro et al. [28] who reported that the level of this fatty acid declined with the introduction of palm kernel meal in the diet, thus allowing for a reduction in the levels of CLA in the meat. As such, it can be suggested that the reason for the higher CLA level in the ST muscle is the inclusion of corn in the PKC diet. The level of ΣPUFA n-3 decreased gradually in the 5% corn and 10% corn compared with the 0% corn. The high PUFA content, including non-essential fatty acids, namely palmitoleic (16: 1) and oleic (18: 1) acids in the mutton should make the meat more attractive to health-conscious consumers. Linolenic acid originates from dietary lipids and could enable the endogenous synthesis of long-chain PUFA by elongase and desaturase activities. This probably is the result of the decreased biohydrogenation activity in the rumen which increases PUFAs available for gut absorption and tissue deposition [32]. The n6:n3 ratio is a significant indicator of the nutritional value of meat since it affects risk factors linked to the development of cancer and heart disease and the formation of thrombi or clots that can cause heart attacks [33]. The type of feeding regimens used in beef cattle production can impact the level of essential fats in red meat due to the differences in the fatty acid composition of the diet [34].

## Conclusion

The findings of this study indicated that the inclusion of corn in the PKC-urea straw diets of Dorper lambs contributes to a significant increase in PUFA concentrations in the SS, ST and LL muscles. The modified deposition and distribution of body fat indicate that PUFA (from corn) could be a repartitioning factor for carcass fat in lambs.

## Methods

### Animal care and diets

The experimental procedures conducted on the animals adhered to the standards of the Institutional Committee on Animal Use Ethics (Approval No. R064/2016). Twenty-seven Dorper lambs (9 animals per treatment) with an average live weight of about 15 ± 0.59 kg were purchased from a local farm in Selangor and randomly assigned and tested under three treatment modes. The lambs were allocated in individual pens equipped with feeders and drinkers. They were fed a basal diet consisting of PKC and urea-treated straw. The dietary treatments were: Treatment 1 (T1) – (0% corn and 75.3% PKC) as a control diet; Treatment 2 (T2) - (5% corn and 70.3% PKC, and Treatment 3 (T3) - (10% corn and 65.3% PKC) (Table 7), the corn substituting partially the PKC. The urea treated rice straw was given at 20% of the total diet on dry matter bases. The three diets were formulated to be isonitrogenous and isocaloric according to feeding standards of [29]. The lambs were allowed 20 days adjustment period followed by 120 days on the experimental diets. The lambs were kept individually and fed twice a day at 8:00 h and 16:00 h with the urea treated straw and concentrate given at a ratio of 20: 80.

**Table 7:** Ingredients and chemical compositions of different inclusion of corn into palm kernel cake – urea rice straw treated (DM basis)

Ingredients	Corn		
	T1 (0%)	T2 (5%)	T3 (10%)
Rice straw Urea treated	20	20	20
PKC	75.3	70.3	65.3
Protected fat (Megalac)	3	3	3
Corn	0	5	10
CaCO_3_	1	1	1
NaCl	0.5	0.5	0.5
Vitamin premix	0.2	0.2	0.2
Total	100	100	100
Chemical composition			
DM	91.78	91.66	91.55
Ash	13.80	12.72	12.74
OM	86.19	87.27	87.26
CP	15.42	14.88	14.09
EE	5.3	5.1	4.33
CF	26.6	24.50	20.83
NDF	62.36	60.06	55.66
ADF	45.60	40.96	37.30
ADL	6.56	6.10	5.43
Hemicellulose	16.76	19.10	18.36
Cellulose	39.03	34.86	31.86
NFE	40.44	41.11	48.39
ME MJ/Kg DM	7.36	8.23	8.92

Vitamin premix; Vitamin A: 10,000,000 IU; Vitamin E: 70,000 IU; Vitamin D: 1,600,000 IU; *DM* Dry matter, *OM* Organic matter, *CP* Crude protein, *EE* Ether extract, *CF* Crude fiber, *NDF* Neutral detergent fiber, *ADF* Acid detergent fiber, *ADL* Acid detergent lignin, *NFE* Nitrogen free extract, *ME* Metabolizable energy

### Slaughtering and sampling procedures

At the end of the 120-day trial period, the lambs were slaughtered according to the standard protocol of the MS 1500:2004 at the Meat Science Laboratory, Department of Animal Science, Faculty of Agriculture, Universiti Putra Malaysia. They were fasted for 12 h with free access to drinking water, transported to the abattoir, allowed to rest, and then weighed before slaughter. The slaughter was performed by a certified and highly experienced technician with a sharp knife. The animals were slaughtered by severing the jugular vein, without being stunned, with a razor sharp knife. In this study, sustained absence of corneal reflex and rhythmic breathing were strictly monitored and checked to ensure that each individual lamb was dead prior to further processing and sampling. The carcasses were shifted to cold room and kept for 24 h at 4 °C. The pH and color of SS, LL*,* and ST muscles were taken at 45 min and 24 h after post mortem. Samples of SS, LL*,* and ST muscles from the right half of each carcass were stored at − 80 °C for fatty acids analysis.

### Meat quality measurements

The meat quality assessments were based on muscle color, water holding capacity, shear force, and pH for three muscles (SS, LL, and ST muscles). Samples were allowed to thaw at 4 °C overnight before analysis. Muscle slices of 1.5 to 2 cm thickness were left to bloom for 30 min at room temperature. Color measurements were documented in triplicate for each sample at randomly chosen positions and stated by the coordinates lightness index (L*), redness index (a*), and yellowness index (b*) of the CIELab colorimetric space [35]. Drip-loss samples of approximately 20 g taken at 0 h *postmortem* was trimmed and weighed and placed into vacuum bags before labeling, vacuum packed (98%) using a chamber Vacuum, and stored at 4 °C. After 24 h *postmortem*, drip loss was computed as the percentage of weight change [36]. The samples for cooking loss used frozen meat which was allowed to cool to room temperature. The thawed samples were each weighed and registered as first weight, kept in polyethylene bags, and inserted in a hot water bath (80 °C) until the inner temperature reached 78 °C. The cooking continued for another 15 min and the samples then removed from the water bath. They were counterweighed to room temperature and then removed from the bags, blotted dry without squeezing, and weighed again. The same muscle samples utilized to determine cooking loss were also used for assessing tenderness. Sample preparation was followed method [37]. Three replicate blocks were cut parallel to the side of the muscle fibers from each sample and each block sheared in the center at right angles to the longitudinal orientation of the fibers. Shear force was the average peak positive force value for all individual sample blocks. The pH of the muscles was measured at 24 h post mortem according to [29] and 0.5 g of the samples crushed with porcelain mortar and pestle with continuous liquid nitrogen flushing. The pH meter (Mettler Toledo, AG 8603, Switzerland) was calibrated with pH 4.0 and pH 7.0 buffers before use. Each pulverized sample was homogenized for 20 s with 10 mL of phosphate buffered saline to avoid continuing glycolysis. The pH of the resultant homogenate was measured by the electrode attached to the pH meter.

### Procedure for fatty acids analysis of meat

The method described by [38] was used for the extraction of fatty acids from the samples and capillary column (60 m, 0.25 mm ID, 0.20 μm film thickness) in a Gas Liquid Chromatography (6890 N, Agilent Technologies, USA). Approximately 1 g of feed and meat (3 replicates per sample) was weighed into a falcon tube and mixed with about 5 ml chloroform/methanol (2:1, v/v). The tube was closed and shaken vigorously and 2.0 ml of normal saline added to assist separation, then vigorously shaken again and vortexed for 5 min followed by centrifuging at 3000 g for 10 min at 24 °C. The top layer containing the non-lipid contaminants was poured out and the lower layer collected in a round-bottom flask and evaporated using a rotary evaporator at 70 °C. The total lipid extract was re-diluted with 5 ml of fresh chloroform-methanol (2:1, v/v) and transferred into a screw-capped methylation tube. About 2 ml of 0.66 N methanolic potassium hydroxide was added to saponify the lipid sample. Nitrogen gas was used to flush the methylation tube which was then placed in a water bath for 10 min. Trans-esterification was initiated by adding 2 ml of 14% BF3 to the fresh mixture and heated for 20 min. Then, 6 ml of distilled water and 4 ml of petroleum ether (boiling point 40–60 °C) were added, vortexed for 60 s, and centrifuged at 3000 g for 10 min to enhance phase separation. The top petroleum phase was removed and placed in another test tube and washed with 1 ml of distilled water to eliminate residual BF3. The top phase of this test tube was carefully collected into a second test tube and dehydrated with 0.5 g anhydrous sodium sulphate. Finally, the FAME was collected into a 4 ml vial, and stored at 4 °C for further analysis. The atherogenic index (AI), desirable fatty acids (DFA) and the ratio of hypocholesterolemic and hypercholesterolemic fatty acids (HH) were calculated according to [39] by following;

$$ AI=\frac{\left(C12:0+4\times C14:0+C16:0\right)}{\left[\sum MUFA+\sum \left(n-6\right)+\sum \left(n-3\right)\right]} $$

$$ DFA=\left( unsaturated\ fatty\ acids+C18:0\right) $$

$$ HH=\left(\mathrm{C}18:1\mathrm{n}-9+\mathrm{C}18:2\mathrm{n}-6+\mathrm{C}20:4\mathrm{n}-6+\mathrm{C}18:3\mathrm{n}-3+\mathrm{C}20:5\mathrm{n}-3+\mathrm{C}22:5\mathrm{n}-3+\mathrm{C}22:6\mathrm{n}-3\right)/\left(\mathrm{C}14:0+\mathrm{C}16:0\right) $$

### Statistical analysis

The data were subjected to a one-way ANOVA as a completely randomized design utilizing the GLM procedure of SAS version 9.4. The means differences were compared using Duncan multiple range tests with significant differences being declared at a probability < 0.05. The statistic model of corn level differences among groups were assessed using the following model:

$$ {\mathrm{Y}}_{ij}=\upmu +{\upalpha}_i+{e}_{ij}, $$

in which Y_*ij*_ = dependent variable; μ = overall mean; α_*i*_ = The fixed effect is for inclusion level of corn (5 and 10%); and *e*_ij_ = experimental error assumed to be NID with (0, *σ*^*2*^ *e*).

## Data Availability

Availability of data and materials used and analyzed during this study is available from the corresponding author on reasonable request.

## Abbreviations

a*: Redness index
b*: Yellowness index
L*: Lightness index
LL: *longissimus lumborum* muscle
MUFA: Monounsaturated fatty acids
PKC: Palm kernel cake
PUFA: Polyunsaturated fatty acids
SCD: Stearoyl-CoA desaturase
SFA: Saturated fatty acids
SS: *supraspinatus* muscle
ST: *semitendinosus* muscle
